# Deep Learning Integration in Optical Microscopy: Advancements and Applications

**DOI:** 10.1002/jemt.70112

**Published:** 2026-01-04

**Authors:** Pottumarthy Venkata Lahari, Sagnika Dutta, H. Deeksha, Samreen A. Patel, Budheswar Dehury, Nirmal Mazumder

**Affiliations:** ^1^ Department of Bioinformatics Manipal School of Life Sciences, Manipal Academy of Higher Education Manipal India; ^2^ Department of Biophysics Manipal School of Life Sciences, Manipal Academy of Higher Education Manipal India

**Keywords:** deep learning, neural networks, optical imaging, optical microscopy

## Abstract

Optical microscopy is a cornerstone imaging technique in biomedical research, enabling visualization of subcellular structures beyond the resolution limit of the human eye. However, conventional optical microscopy faces challenges such as optical aberrations, diffraction‐limited resolution, low signal‐to‐noise ratio (SNR), and poor contrast. The exponential growth of bioimaging data further underscores the need for advanced computational tools. Deep learning (DL) is a subset of machine learning that has emerged as a transformative approach to address these limitations, offering enhanced precision, reduced manual intervention, and diminished reliance on domain‐specific expertise for image reconstruction, enhancement, and analysis. This review explores the integration of DL into optical microscopy, focusing on key applications including image classification, segmentation, and computational reconstruction. We examine prominent DL architectures such as convolutional neural networks (CNNs), U‐Nets, residual networks (ResNets), and generative adversarial networks (GANs)—and their role in advancing diverse microscopy modalities. These frameworks enhance image quality, improve quantitative analysis, and democratize access to high‐performance microscopy. Additionally, we discuss persisting challenges, including the demand for large, annotated datasets, dynamic sample variability, model interpretability, and potential data biases. Collectively, DL is poised to revolutionize optical microscopy, shaping its future developments in biomedical imaging.

## Highlights

- Aberrations, diffraction limits, low signal‐to‐noise ratio, and poor contrast are key obstacles which hinder high‐quality imaging.
- Deep learning enhances image reconstruction, segmentation, and analysis by reducing manual effort and expert dependence, using models like CNNs, U‐Nets, ResNets, and GANs.
- While DL improves image quality and accessibility in microscopy, challenges remain, including the need for large annotated datasets, variability in biological samples, and concerns about model interpretability and bias.

## Graphical abstract

It explores the integration of DL into optical microscopy, focusing on key applications including image classification, segmentation, and computational reconstruction.

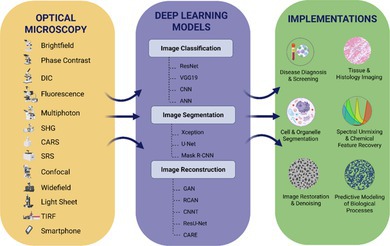

## 1 Introduction

For more than 400 years, optical microscopy has led to numerous ground‐breaking discoveries in a variety of natural disciplines (Palounek et al. 2024). Optical microscopy employs light to visualize details that are beyond the eye's resolving power, creating contrast through reflection, absorption, polarization, and fluorescence. It is crucial for biomedical research because of its great spatial resolution and non‐invasiveness, serving as a vital imaging tool (Zhang, Hu, et al. 2023). Several optical microscopy techniques discussed here include total internal reflection microscopy (TIRF), widefield fluorescence microscopy, second harmonic generation (SHG), confocal, brightfield, fluorescence, differential interference contrast, phase contrast, multiphoton, coherent anti‐Stokes Raman scattering, stimulated Raman scattering, and light sheet microscopy.

Despite its strength, optical microscopy has some significant drawbacks, such as blurring, poor lateral and axial resolution, and diminished contrast at high magnification (Kaderuppan et al. 2020). These limitations are prominent during live cell imaging, where a poor signal‐to‐noise ratio is a consequence of lowering the light exposure to minimize the photobleaching and phototoxicity (Ichita et al. 2025; Qu et al. 2025). For visualizing transparent biological samples, we usually stain or label them, which can lead to permanent damage to the samples (Ichita et al. 2025; Liu et al. 2025). Meanwhile, on the other side, microscopes such as DIC that are based on label‐free techniques don't provide quantitative intensity information. Manual analysis of microscopy data is often time‐consuming, labour‐intensive, expensive, and might lead to inconsistencies, which can impact the dynamic biological research (Frangos et al. 2025; Lv et al. 2025; Qu et al. 2025). Machine learning's (ML) rapid rise in recent years has been fuelled by the expansion of data and improvements in computing power (Hanna et al. 2025). Artificial intelligence (AI) includes machine learning as a subfield. Artificial intelligence aims to build computer models that exhibit “intelligent behaviors” like those of people. Deep learning techniques, which use a cascade of many layers of nonlinear processing units to extract and transform information, use the output from the previous layer as input for each new layer (Amin et al. 2024). A neural network is designed based on the structure of the human brain and it trains the computer to imitate human reasoning. Deep learning has made impressive progress in numerous fields (Meitei et al. 2023).

Employing deep learning in optical microscopy is a major development that goes beyond conventional image processing techniques (Liu et al. 2025). They work exceptionally well for denoising and noise correction, especially in severe low‐SNR conditions that arise during low‐light imaging required for sample preservation. Additionally, deep learning is essential for automating numerous types of analysis tasks, such as tracking, cell segmentation, and quantitative analysis, which speeds up research and overcomes the drawbacks of manual approaches for big datasets (Frangos et al. 2025; Ichita et al. 2025).

Deep learning has become a robust to overcome the challenges in the field of optical microscopy. The drawbacks of various microscopy techniques such as noise, low contrast, diffraction‐limited resolution, and complex image reconstruction have been addressed by incorporating the useful DL models such as CNNs, U‐Nets and many others to improve the efficiency of tasks such as classification, reconstruction and segmentation. However, the current literature is restricted to either broad overview of DL and its models or a specific task or method and does not convey much about the way DL models complement microscopy. The novelty of this review lies in its problem‐driven approach, highlighting and demonstrating the use of various DL models to mitigate microscopy drawbacks. In contrast to traditional model or task‐centric surveys, this study connects computational solutions with optical obstacles, highlighting successful model‐modality combinations. We also discussed the issues that remain unsolved and how deep learning can influence bioimaging research in the future.

## 2 Types of Optical Microscopy Techniques

### 2.1 Conventional Optical Microscopy

#### 2.1.1 Brightfield Microscopy

The brightfield optical microscope remains a traditional and vital tool in bioimaging because of its straightforward, fast and economical nature (Guti Errez‐Medina 2022). The basic principle of this microscope involves passing incident light through the sample, and this is often provided by a quartz tungsten halogen source. The image is then created by detecting difference in light absorption or scattering. The typical setup includes adjusting the objective lenes focus close to the surface (Zhu et al. 2020). The scattered light by sample (E_s_) and the background signal (E_b_) (which is comprised of direct transmitted light and reflected light from the microscope lens) merge to produce detected signal. To differentiate the different elements in the raw image, the background signal needs to be eliminated. The intrinsic imaging contrast is significantly affected by this bright background. The contrast of the image is extremely variable between the sample structure and the surface (Guo et al. 2024; Zhu et al. 2020).

**FIGURE 1:**
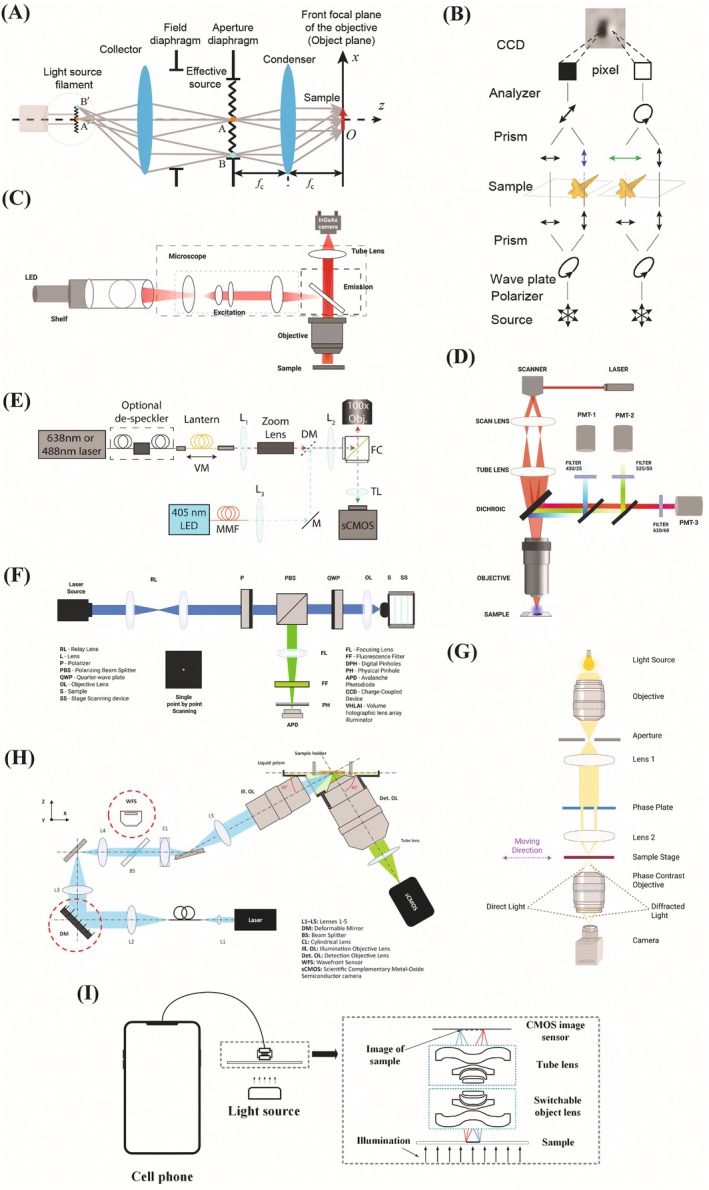
Schematic diagram of optical microscopy techniques. (A) Brightfield (reused with permission from Ma et al. (2023)), (B) DIC (reused with permission from Hu et al. (2020)), (C) Widefield (reproduced with permission from Cai, Zhu, et al. (2020)), (D) Multiphoton (E) TIRF (reused with permission from Husain et al. (2024)), (F) Confocal (reproduced with permission from Suresh et al. (2025)), (G) PCM (reproduced with permission from Zhang, Wang, et al. (2022)), (H) LSFM (reused with permission from Park et al. (2023)), and (I) Smartphone‐based (reused with permission from Wan and Tao (2021)).

This technique has a major drawback for imaging live cells, as it requires fixation, which leads to cell death (Salem et al. 2021). Although unstained samples frequently have low contrast, brightfield microscopes can now provide crisper, three‐dimensional images of label‐free samples without the need for specialized equipment, owing to modern techniques that use Köhler illumination and digital image processing (Guti Errez‐Medina 2022). The resolution of standard brightfield microscopy is limited to nearly half the wavelength of light, regardless of significant optical optimization (Guo et al. 2024). The integration of deep learning is a common advancement as it provides efficient and accurate segmentation of live images, which is significant in optimizing the data extraction as well as information generation from high‐throughput biological experiments. Manual segmentation is too tedious and prone to error (Salem et al. 2021). Figure 1 describes various types of microscopy techniques.

#### 2.1.2 Phase Contrast Microscopy (PCM)

PCM is a classic label‐free imaging method that makes transparent, unstained specimens (e.g., living cells) visible by converting minute optical phase shifts into intensity contrast. In a PCM, a hollow cone of illumination is produced by a condenser annulus, so that most light (the “background” or zeroth‐order beam) passes through the sample undeviated while a small portion is diffracted by structures of varying refractive index (Nguyen et al. 2022). The refractive‐index variations in the sample thus introduce a relative phase delay in the diffracted wavefront compared to the unscattered background (Nguyen et al. 2022; Nienhaus et al. 2023). A key component is a ring‐shaped phase plate in the objective's back focal plane: in positive phase contrast systems, this plate retards the background light by +π/2 (a quarter‐wave) relative to the scattered light (Ghosh and Agarwal 2023; Jayakumar and Ahluwalia 2025). When the two wave components recombine at the image plane, they interfere—for weak phase objects the resulting intensity is approximately proportional to the original phase shift (Jayakumar and Ahluwalia 2025; Nguyen et al. 2022). In other words, the phase‐shifted wave and the retarded background light produce constructive or destructive interference so that even tiny optical‐path differences (caused by refractive‐index changes) appear as brightness variations in the image (Jayakumar and Ahluwalia 2025; Nguyen et al. 2022). Thus PCM translates otherwise invisible phase gradients into clear intensity contrast (Jayakumar and Ahluwalia 2025; Nienhaus et al. 2023).

In practice, the condenser annulus and objective's phase ring must be conjugately aligned so that undiffracted light is focused onto the phase plate (yielding an even background), while scattered light from the specimen largely bypasses it (Nienhaus et al. 2023). Under these conditions, phase changes at specimen boundaries appear as bright or dark contours in the image (Nienhaus et al. 2023). The phase‐to‐amplitude conversion in PCM also introduces characteristic artifacts. In particular, the so‐called “halo” effect—a bright or dark rim around phase objects—is a well‐known consequence of the phase plate design (Nienhaus et al. 2023). Likewise, uneven illumination (“shade‐off”) can occur when phase contrast conditions are not perfectly met. These imperfections stem from the finite ring width and nonideal π/2 shift, which prevent a perfectly linear translation of phase into intensity. Modern approaches often use computational methods (e.g., image restoration or deep learning) or improved optics to mitigate these artifacts and extend the useful field of view (Jayakumar and Ahluwalia 2025; Nienhaus et al. 2023).

#### 2.1.3 Differential Interference Contrast (DIC)

DIC, a label‐free brightfield optical microscope, remains a significant tool in cell biology, which can observe vulnerable structures in unstained and living samples with low toxicity. The fundamental principle of this microscope is that it converts an object's intrinsic phase difference, which comes from the local variation in thickness or refractive index, into visible amplitude variations by coherent light interference (Pan et al. 2024). The main mechanism used in the DIC optical system is shear interferometer (Zhang, Sarollahi, Luckhart, et al. 2024). It consists of a Nomarski prism, which separates the polarized light into two orthogonally polarized beams (e‐ and o‐ waves) (Kale et al. 2024; Zhang, Sarollahi, Luckhart, et al. 2024). The spatial separation by these two beams is less than the width of the point spread function. As it ensures that both fields undergo the same degradation as they travel through the sample, covering up the non‐uniformity, this minimal lateral shear is essential. To form the interference, the beams are recombined by the analyzer (the second prism) once passing through the sample, which is typically within the range of 1 μm or less. The resultant image represents the specimen's gradient‐phase (**∇**_**x**_ **Φ**) (Pan et al. 2024; Zhang, Sarollahi, Luckhart, et al. 2024). This ability provides minimal toxicity, which is especially useful for time‐lapse imaging and when paired with Quantitative Phase Imaging (QPI) using integration methods like Hilbert transforms, which facilitates the measurement of dry mass and density (Kale et al. 2024). This technique is highly helpful for applications that need very little sample disturbance, such as examining the shape of sperm cells (Noy et al. 2023) or structural analysis of platelets in hematology (Kempster et al. 2022). Despite these advantages, DIC image processing faces lots of analytical limitations. DIC images are basically qualitative, two‐dimensional, and hampered by an undesirable shadow effect (Kempster et al. 2022; Noy et al. 2023). Recently, the integration of DIC along with the DL has been used to solve the issue that arises during the processing of images (Pan et al. 2024).

### 2.2 Fluorescence Microscopy

The principle of fluorescence microscopy involves a fluorophore absorbing a single photon of light of short wavelength and emitting a longer wavelength, which is then easily differentiated from the excitation light. This difference between the short excitation wavelength and the longer emission wavelength is termed Stokes shift, which forms the basis of contrast in fluorescence systems. The utilization of dichroic mirrors and emission filters can effectively separate the excited light from emitted light to detect only the fluorescent signal, thus ensuring only labeled parts of the image fall on the detector. As the transition of the fluorophore from excitation to emission occurs in a few nanoseconds, this decay duration can be measured and used as an additional contrast source in fluorescence lifetime imaging microscopy (FLIM) (Luu et al. 2024). Historically, the necessity of the fluorophore restricted the use of this technique to samples with autofluorescence. However, with the development of fluorescent stains and fluorescently labeled antibodies, and eventually the introduction of green fluorescent proteins (GFP) and their use as a genetic tag, this technique could be used with non‐fluorescent samples. The discovery of novel fluorescent markers has led to rapid advancements in fluorescence imaging (Hickey et al. 2022). An advanced technique called optical sectioning structured illumination microscopy (OS‐SIM), was developed to image thick, deep tissue samples. It has led to high clarity images by removing unwanted and out‐of‐focus contributions from above and below the focal plane (Chen et al. 2023).

#### 2.2.1 Confocal Microscopy

Confocal microscopy works on the fundamental principle of both illumination and detection being focused on the same spot, which moves all over the sample to construct the image. In confocal microscopy, even when the complete field of view is illuminated, light from outside the focal plane is blocked, which reduces haziness and allows optical sectioning. Marvin Minsky developed the technique in 1955 and patented it in 1957 (Eissa et al. 2024). In a standard confocal setup, this is achieved by a laser focused onto a single spot and the spot is scanned across the sample in a raster pattern with the help of oscillating mirrors. As excitation and emission occur in a few nanoseconds, the mirror displacement is negligible, meaning the fluorescence follows the same optical path back to the laser. This emission is then separated from the excitation light with a spectral‐specific reflector called a dichroic mirror and is focused into the pinhole, allowing in‐focus light alone to reach the detector (Amos 2025). A variation called spinning disk confocal microscopy (SDCM) scans many points on a sample simultaneously via a spinning disk with numerous pinholes while also rejecting out of focus light from fluorophores hitting the sample, which is then integrated by a camera to create the image. Because parallelized acquisition reduces the peak illumination power density for the numerous spots the SDCM uses, it can accomplish fast imaging rates with less photobleaching or phototoxicity (Halpern et al. 2022). In both in vivo and ex vivo approaches, confocal microscopy is an invaluable instrument for non‐invasive or rapid quasihistological tissue examination in clinical environments (Braghiroli et al. 2022).

#### 2.2.2 Total Internal Reflection Fluorescence (TIRF) Microscopy

TIRF is an optical method wherein fluorophores are excited in a very thin optical section. The fundamental principle of this technique is that an electromagnetic field known as the evanescent wave, which has the same frequency as the excitation light, is produced in the liquid at the solid–liquid interface when excitation light is fully internally reflected in a transparent solid. Only fluorescent molecules within a few hundred nanometers of the solid are effectively activated because the intensity of the evanescent wave exponentially decreases with distance from the solid's surface. In TIRF microscopy, sample is generally illuminated by a laser beam placed at an angle greater than critical angle (Dzikonski et al. 2025; Fish 2022). Therefore, TIRF microscopy's primary benefit is that its high axial resolution guarantees high‐contrast fluorescence images; yet its biggest drawback is the sample's limited thickness under examination (Mochalov et al. 2025). RNA folding, transcription, DNA replication, macromolecular complex formation, and other unsynchronized intra‐ and intermolecular interactions between fluorescently labeled biological molecules are all studied in real time using TIRF (McCluskey and Dekker 2023).

For single‐shot TIRF illumination from multiple azimuthal directions simultaneously, the photonic lantern, a tapered waveguide that serves as a highly efficient 1 × *N* fiber splitter, distributes light from a single multimode input to multiple output cores resulting in an artifact‐free, uniform TIRF excitation (Husain et al. 2024). A similar surface detection optical technique is Supercritical Angle Fluorescence (SAF) and Supercritical Angle Raman (SAR) spectroscopy and microscopy. These techniques detect light which is emitted or scattered by the molecules at bulk or near surface. The bulk molecules emit light at subcritical angles whereas the surface molecules emit at supercritical angles, both of which are recorded in separate channels thereby allowing us to study surface and bulk processes simultaneously. These in turn, enable precise detection of changes or molecular events occurring at the surface (Münch et al. 2024).

#### 2.2.3 Widefield Fluorescence Microscopy (WF Fluorescence)

WF fluorescence microscopy is a simple microscopy technique wherein a parallel beam of light illuminates a sample simultaneously as a whole to excite the fluorophore. It enables us to view all the specimens' resulting fluorescence at once, enabling quick and easy imaging (Wang and Yunyan 2021). There is an imaging array which subsequently gathers the fluorescence. WF microscopy provides greater spatial and temporal resolution (Peterkovic et al. 2025). WF microscopy achieves greater time resolution by simultaneously capturing entirety of the field of view, unlike point‐scanning techniques that rely on external X‐Y scanning devices to sequentially identify surface points and build two‐dimensional images. Additionally, the process is straightforward and simpler to operate because neither beam focusing nor point‐by‐point excitation are required (Wang, Chen, et al. 2023). WF fluorescence imaging may attain kilohertz (kHz) frame rates across a broad (centimeter‐scale) field‐of‐view, but a lack of depth information imposes major limits on many applications that require optical sectioning and quantification abilities (Zhou et al. 2022).

### 2.3 Nonlinear Multiphoton Microscopy

Nonlinear Multiphoton microscopy, which includes two‐photon excited fluorescence microscopy (2PFM) and three‐photon excitation microscopy (3PEF), is a technique developed for non‐invasive imaging of biological samples. It allows deep tissue visualization with very high optical resolution (Buttolph et al. 2023; Lu et al. 2025). The term “nonlinear” means that the sample requires interaction of multiple photons at the same time to get excited, as opposed to a direct linear relationship. In single photon microscopy, a photon with sufficient energy can send the fluorophore to the excited state and on returning to ground state, it emits a photon of a longer wavelength. In two‐photon microscopy, longer wavelengths are used, meaning each photon has lesser energy thus requiring two photons simultaneously to reach the same excitation state (Borile et al. 2021). The probability of the two‐photon excitation process is directly proportional to the square of photon density of the excitation photons. Due to this, multiphoton absorption and emission of fluorescence is restricted to the objective lens's laser focal spot, thus reducing unnecessary light scattering in tissues and more clarity (Amos 2025). This has led to new heights in research as even when imaging the same fluorophores, two‐photon fluorescence microscopy obtains a penetration depth two to three times greater than that of one‐photon microscopy (Xu et al. 2024).

#### 2.3.1 Second Harmonic Generation (SHG) Microscopy

SHG is a non‐invasive imaging technique which explores the physiological structures without the use of an exogenous label (Zhang, Lin, et al. 2023). The main principle of SHG deals with the interaction of photons with matter. SHG, a nonlinear optical process results in a single photon with a doubling frequency (2ω) when two photons with equal energy and frequency (ω) interact in a non‐centrosymmetric medium (Zhang, Zhang, Feng, et al. 2024). A material under an optical electric field contains both linear and nonlinear events, which can be thought of as springs in a Lorentz model. The positively charged nucleus and negatively charged electron cloud are attracted to one another by Coulombic forces. An oscillating electric dipole moment is produced when the spring is stretched by electromagnetic radiation. A linear optical effect occurs when the induced dipole moment is proportional to the electric field. However, nonlinear components of the dipole moment become prominent at greater field strengths, leading to phenomena like SHG (Zhang, Tan, et al. 2023). The laser in an SHG instrument usually works in the near‐infrared I (NIR‐I) range as the absorption loss is minimum. When the laser employs longer wavelengths, scattering is limited and penetration depth increases but the spatial resolution is compromised thereby resulting in low SHG signals, hence shorter excitation wavelengths are preferred (Aghigh et al. 2023). The specimens and structures that are studied using SHG must either be stained with specific dyes or non‐centrosymmetric so that they respond well to this technique and generate good quality images (Dudenkova et al. 2019). Since the samples are dyed, SHG applications are restricted to few structures but is also the reason for sharp contrast images resulting due to highly specific signals. In comparison to fluorescence, SHG generates an instantaneous signal free from photobleaching. Hence, SHG has emerged as a potent technique for multimodal high spatial resolution optical imaging (Aghigh et al. 2023).

#### 2.3.2 Coherent Raman Scattering (CRS) Microscopy

CRS microscopy refers to a class of nonlinear vibrational imaging techniques, primarily coherent anti‐Stokes Raman scattering (CARS) and stimulated Raman scattering (SRS), in which multiple synchronized lasers excite molecular vibrations coherently (la De Cadena et al. 2022; Zhitnitsky et al. 2024). In CRS, a pump laser at frequency ω^p^ and a Stokes laser at ω_s_ are overlapped in the sample focal volume, and if their difference Δω = ω^p^–ω_s_ matches a molecular vibrational frequency, it drives coherent Raman transitions (Li et al. 2020; Ranjan and Sirleto 2024). In CARS, the pump and Stokes photons induce a third‐order polarization that emits a new photon at the anti‐Stokes frequency (S. Li et al. 2020; Zhitnitsky et al. 2024). Because this anti‐Stokes photon is blue‐shifted relative to the pump, it can be filtered spectrally to yield label‐free chemical contrast, and the nonlinear excitation inherently provides intrinsic 3D optical sectioning (no pinhole needed) (Zhitnitsky et al. 2024). In SRS, by contrast, the resonance causes energy to be transferred from the pump beam to the Stokes beam: this appears as a small loss in pump intensity and a corresponding gain in Stokes intensity when on vibrational resonance (Li et al. 2020; Ranjan and Sirleto 2024). The coherent driving of many identical molecular oscillators means that CRS signals are orders of magnitude stronger than spontaneous Raman scattering (la De Cadena et al. 2022; Ranjan and Sirleto 2024). SRS thus provides a strictly resonant, background‐free contrast (since it is detected as an intensity modulation rather than a new frequency), whereas CARS signals inherently include a non‐resonant electronic background contribution (Li et al. 2020). Efficient CRS imaging requires ultrafast, tuneable laser pulses and careful phase matching in the focus; typically picosecond pulses are used to balance spectral resolution and nonlinear efficiency (Li et al. 2020; Zhitnitsky et al. 2024). Modern CRS systems may use narrowband excitation (targeting one Raman line at a time) or broadband/multiplex excitation (la De Cadena et al. 2022).

### 2.4 Light Sheet Fluorescence Microscopy (LSFM)

LSFM works by selectively illuminating only a single plane of the specimen at a time with a thin “sheet” of laser light, while collecting the emitted fluorescence at a right angle using a wide‐field detection objective (Horejs 2024). In practice, a laser beam is shaped into a thin waist—often using a cylindrical lens to form a Gaussian‐profile sheet, though engineered beams such as Airy or Bessel beams are also employed, that overlaps the focal plane of the detection optics (Daetwyler and Fiolka 2023). Only fluorophores in this focal plane are excited, so out‐of‐focus regions remain largely dark; this gives LSFM true optical sectioning and very sharp, blur‐free images of each plane (Daetwyler and Fiolka 2023). Because only one slice of the sample is illuminated at a time, photobleaching and phototoxicity are drastically reduced compared to epi‐illumination methods (Daetwyler and Fiolka 2023; Horejs 2024). To build a 3D image, the light sheet (or the sample itself) is translated along the imaging axis to sweep through the volume, and successive planes are captured on a camera (Daetwyler and Fiolka 2023). Since each plane is imaged in a single camera exposure, LSFM has a high “spatial duty cycle” and can acquire volumes much faster and with lower light intensity than point‐scanning techniques like confocal microscopy (Daetwyler and Fiolka 2023; Horejs 2024). In sum, the orthogonal arrangement of illumination and detection and the use of a thin planar excitation beam enable LSFM to perform rapid, high‐resolution volumetric imaging of large or live specimens with dramatically reduced photodamage (Daetwyler and Fiolka 2023; Tang et al. 2025).

### 2.5 Smartphone‐Based Microscopy

Smartphone‐based microscope represent a major development in Point‐of‐Care (PoC) diagnostics, converting conventional mobile phones into compact, affordable, and easy‐to‐use imaging devices (Huang et al. 2024). The fundamental principle of smartphone microscopy involves transforming a smartphone's in‐built camera system, which is based on an infinite conjugate system designed for detecting distant objects and generating minimized images, into high magnification depends on lens setup. To observe micron‐scale structures, external lenses have to be integrated as part of this important conversion. The extremely sensitive Complementary Metal‐Oxide‐Semiconductor (CMOS) sensor onto the smartphone gets the enlarged image directly from the external lens module, which usually acts as the objective. The basic features of the sensor, like its high pixel count and small pixel size (e.g., 1.4 μm), are the significant variables in influencing the final image quality along with possible resolution. The optical magnification (M) is calculated by dividing the internal camera focal length (f1) with the external lens's focal length (f2), which is theoretically expressed as M = f1/f2 (Huang et al. 2024). The external optical parts, illumination sources, and the filters are usually mounted in lightweight, customized opti‐mechanical attachments that are usually 3D‐printed and offer the exact alignment necessary for high‐resolution imaging (Leonard et al. 2022; Loretan et al. 2025). Beyond magnification, modern illumination and computational techniques define the operational depth of modern smartphone microscopes. Methods like Smartphone‐based Autofluorescence Microscopy (Smart‐AM), where UV light‐emitting diodes (UV‐LEDs) are employed for oblique illumination, contrast creation often goes beyond the typical brightfield microscope (Huang et al. 2024). DL algorithms are integrated to perform highly precise tasks such as detection and classification. Overall, this microscope provides a handy and affordable substitute for traditional microscopes (Leonard et al. 2022; Trofymchuk et al. 2021).

## 3 Types of Deep Learning Models

**FIGURE 2:**
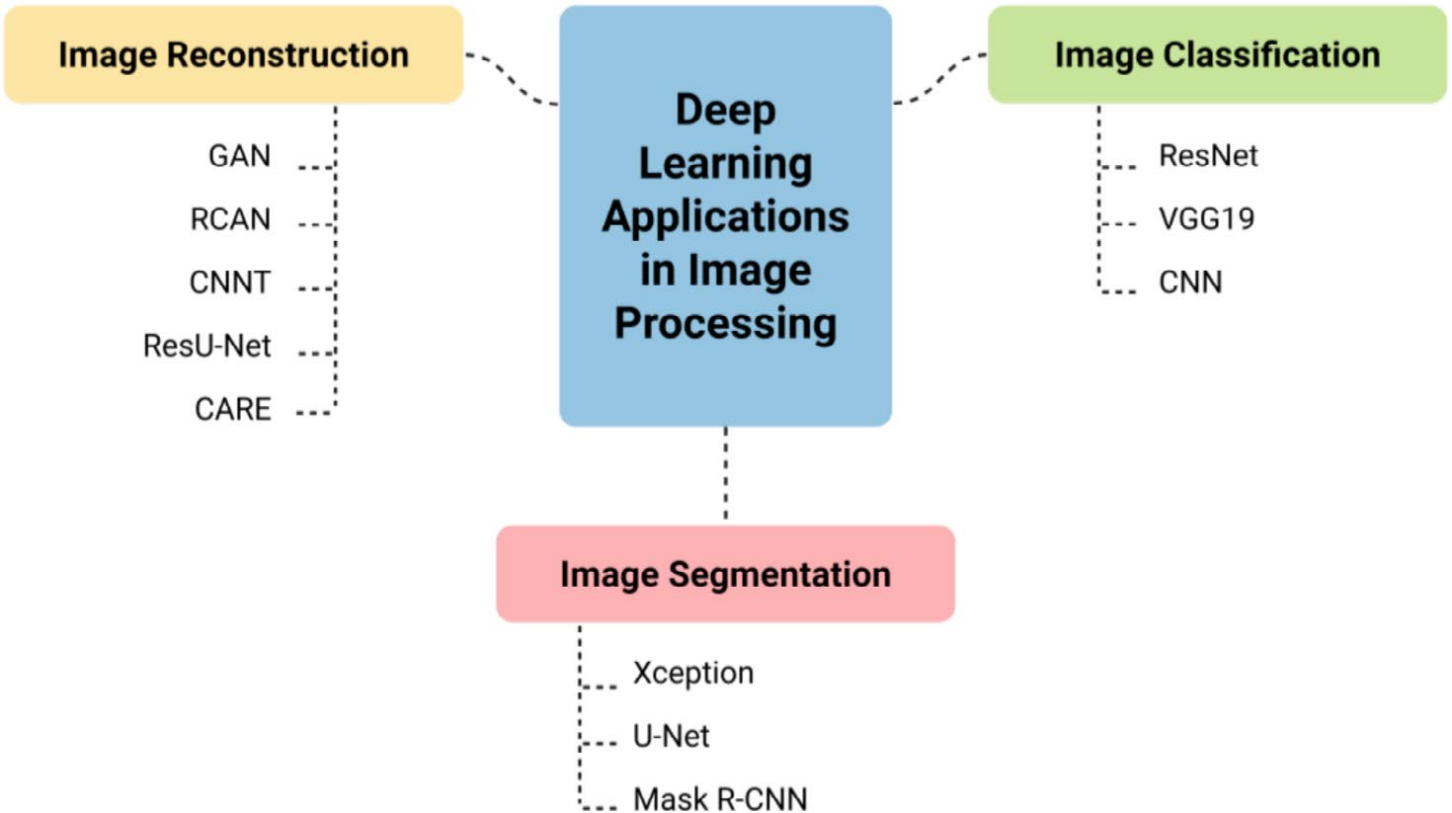
An illustration of how deep learning is applied to image processing in optical microscopy. The applications are grouped into three core tasks—image classification, segmentation, and reconstruction—with relevant neural network models supporting each category.

A branch of machine learning known as “deep learning” is capable of autonomously extracting important features from unprocessed data. DL models are more equipped to handle complicated, high‐dimensional, and nonlinear data than machine learning models (Yun et al. 2024). DL aims to create learning algorithms that accurately reflect the complexities of the human brain, with its roots in the need to simulate cognitive processes (Archana and Jeevaraj 2024). A DL model is structurally made up of several multi‐layer perceptrons. Usually, it has several hidden layers with millions of artificial neurons. Several processing layers are applied to the input data before an intelligent output is generated. The following processing hierarchy then uses the representation of the first hidden layer as input, and so on (Bayoudh 2024). Figure 2 shows the types of DL applied in image processing.

Deep Learning Network (DLN), also known as Deep Neural Network (DNN), is formally defined as a computational architecture with more than one hidden layer (Xie et al. 2025). It significantly differs from that of a Shallow Neural Network (SNN), which typically just has one hidden layer, because of its complex structure and dependence on a vast number of parameters (Bevilacqua et al. 2019). In terms of functionality, DNNs reduce the demand for manually created features by using a built‐in feature learning to automatically extract intricate and abstract features (Bevilacqua et al. 2019; Xie et al. 2025). DNNs perform exceptionally well in predictions, yet because of their complexity, they have lower robustness and are less interpretable. On the opposite side, SNNs have great interpretability and robustness due to their simpler structure and fewer parameters but have poorer prediction accuracy (Xie et al. 2025).

### 3.1 Image Classification

The process of recognizing and classifying items in an image is known as image classification. It is a time‐consuming task but has been automated using DL models which recognize the items in an image based on color, feature or texture and classify based on the same (Bansal et al. 2023). The most popular model used for this task is CNN (Convolutional Neural Networks). Later many models were developed such as GoogleNet, VGG19 which improved the accuracy of this task and reduced overfitting (Cai and Gao 2020). The various models used to perform this task are as follows:

#### 3.1.1 ResNet

One of the topologies of convolutional neural networks (CNNs) is called ResNet, or the “residual network.” ResNet constitutes several layers namely the convolution layer, pooling layer, dropout layer, and fully connected layer (Gilbert et al. 2020; Hasanah et al. 2023). ResNet's extensive success in image classification tasks can be attributed to its ability to train deep networks in a stable and effective manner using residual blocks with shortcut connections (Liao et al. 2025). The primary goal of ResNet design is to address the neural network degradation issue, which implies that the training error rate increases with network depth (Gilbert et al. 2020). The fundamental concept remains the same in all ResNet models despite varying number of layers in different models. The naming convention of the models depends on the number of layers a model constitutes. For instance, ResNet‐50 has 50 layers and so on (Gilbert et al. 2020; Hasanah et al. 2023). Input is fed into the convolution layer; task of the pooling layer is to reduce spatial dimension and the number of parameters and finally classification is done by the fully connected layer (Hasanah et al. 2023). ResNet's increased depth and complexity result in redundant features that lower generalization and performance, even though it addresses deep network training concerns and performs well in image classification. As a result, ResNet‐based models continue to struggle with feature redundancy, and adding a universal feature selection method might enhance their capacity for inference (Liao et al. 2025).

#### 3.1.2 VGG19 (Visual Geometry Group 19)

VGG19 is a 19‐layer convolutional neural network design that resembles VGG16. The Visual Geometry Group in the year 2014 introduced VGG19 model at the University of Oxford (Madhur et al. 2023). Sixteen convolution layers plus Rectified Linear Units (ReLUs), five max pooling layers, three fully connected and dropout layers, and a SoftMax classifier make up the VGG19 (Dey et al. 2021). To maintain spatial resolution, VGG19 employs convolutional layers with batch normalization, ReLU activations (except from the last layer), 3 × 3 filters, stride 1, and padding 1. With increasing filters, max pooling with stride 2 decreases the spatial dimensions across the convolutional layer stacks: 64 → 128 → 256 → 512. After a final pooling layer, the network consists of three fully connected layers that use SoftMax to identify dataset classes (Vignesh et al. 2023). Like any other model, VGG19 also has limitations. Since it is designed with so many layers, it will require an enormous amount of parameters, dataset, memory capacity and immense time to classify the objects during the training phase (Bansal et al. 2023).

#### 3.1.3 Convolutional Neural Network (CNN)

CNNs represent a sophisticated class of deep learning models specifically designed for image analysis. These networks comprise sequential layers that progressively extract more abstract features from input images (Hussain et al. 2025; Mienye and Swart 2024). The convolutional layers employ learned filters to identify local patterns, including edges and textures, while pooling layers serve to downsample feature maps, resulting in hierarchical representations. As the network layers deepen, it becomes adept at capturing increasingly complex structures. Additionally, the concept of weight sharing enhances translation invariance and minimizes the number of learnable parameters. The final layers, typically fully connected or global pooling followed by a classifier, produce predictions for tasks like classification or regression. CNNs are trained end‐to‐end using backpropagation to optimize filter weights based on a loss function. Modern architectures such as ResNet introduce skip connections to enable training of deeper networks. In biomedical imaging, CNNs are widely used for tasks such as disease classification and serve as backbones or feature extractors in segmentation pipelines, particularly when labeled data is limited (Hussain et al. 2025; Zhao et al. 2024). In optical microscopy, CNNs enable feature learning and noise reduction, but they generally require large training sets and may produce artifacts if unconstrained (Li et al. 2024).

### 3.2 Image Segmentation

Image segmentation is the first crucial step in image analysis which is responsible for identifying and isolating the region of interest (ROI) (Saba 2021). Segmentation task includes semantic segmentation, which analyses each pixel and assigns it to an object and decides the layout of objects in the image using spatial information, and instance segmentation goes one step further by differentiating between items that belong to the same category (Luo, Yang, et al. 2024; Wang et al. 2022).

Efficient segmentation is important as it assures the following feature measurements determined exclusively based on relevant regions like material microstructures or lesion border areas (Ryan and Agaian 2025; Saba 2021). Following isolation, image texture is a fundamental concept for quantifying intrinsic properties like regularity and roughness using spatial variation in pixel intensities (Liu and Aldrich 2022). This quantification is important in clinical diagnosis, such as in skin cancer, and in explaining material microstructure, as it provides an outline of the morphology by analyzing the correlation between nearby pixels (Basak et al. 2021; Liu and Aldrich 2022; Saba 2021). To mathematically quantify morphological variances, as well as a critical overview of morphology of materials or cellular components, these textural features are best expressed using Haralick features extracted from Grey‐Level Co‐occurrence Matrix (GLCM) and are recovered from the segmented regions (Ma et al. 2021; Saba 2021).

The various models used to perform image segmentation task are as follows:

#### 3.2.1 Xception

Xception, a modified CNN architecture built for image segmentation employs skip connections and unique convolutional layers. These architectural decisions intend to enhance the gradient flow during training, which aids in the identification of minute features (Kumar et al. 2024). In this model, depth wise separable convolutions (DSC), which perform spatial convolution over each channel separately and later combine them, replace the traditional convolutions. This reliance on DSC leads Xception to achieve a superior performance when compared to other models. Models using Xception have also shown to exhibit better inference time in producing the final output. However, Xception is slower than other models during training by a small margin and can often result in overfitting, necessitating optimisation (Chollet 2017; Gurita and Mocanu 2021).

#### 3.2.2 U‐Net

U‐Net is a specialized CNN with a symmetric encoder–decoder (“U‐shaped”) architecture tailored for pixelwise image reconstruction or segmentation (Pan et al. 2025; Zuo et al. 2022). The encoder (contracting) path applies repeated conv–ReLU blocks and pooling to downsample the image and extract context, whereas the decoder (expansive) path uses upsampling (transpose‐convolutions) followed by conv–ReLU blocks to gradually restore spatial resolution. Crucially, U‐Net uses skip (concatenation) connections that link each encoder layer to the corresponding decoder layer at the same resolution, directly injecting fine spatial details from early layers into the upsampling path. This design allows U‐Net to combine coarse, high‐level context information with precise localization information, yielding sharp, per‐pixel outputs. The final layer is usually a 1 × 1 convolution that maps the decoder's features to the desired number of output channels (e.g., segmentation classes). Like CNNs, U‐Nets are trained end‐to‐end via gradient descent on a pixelwise loss (e.g., cross‐entropy or Dice loss) so that their output maps match ground‐truth label (Pan et al. 2025; Zuo et al. 2022). U‐Nets excel at preserving fine‐grained details and achieving precise segmentation (Krikid et al. 2024). However, they can be computationally intensive and struggle with objects across widely varying scales or complex morphologies (Safarov et al. 2025).

#### 3.2.3 Mask R‐CNN

Mask R‐CNN is a DL model, which integrates pixel‐level segmentation and object detection into a unified framework for instance segmentation (Wang et al. 2021). Through the addition of a branch that creates binary masks for every item recognized, it expands on Faster R‐CNN. The model combines a Region Proposal Network (RPN) that suggests object positions, a backbone CNN (such as ResNet) for extracting features, and RoIAlign to maintain spatial accuracy. It predicts the class, segmentation mask, and bounding box for every Region of Interest (Shu et al. 2020; Wan et al. 2025). It is highly beneficial for applications like cell segmentation in microscopy images, as it can separate overlapping objects (Johnson 2018). The primary drawback of this model is that it uses horizontal bounding boxes (the largest outer rectangle), which generally capture unnecessary background information and thereby decreasing the detection accuracy, particularly when dealing with long, narrow or rotated objects (Wan et al. 2025).

**FIGURE 3:**
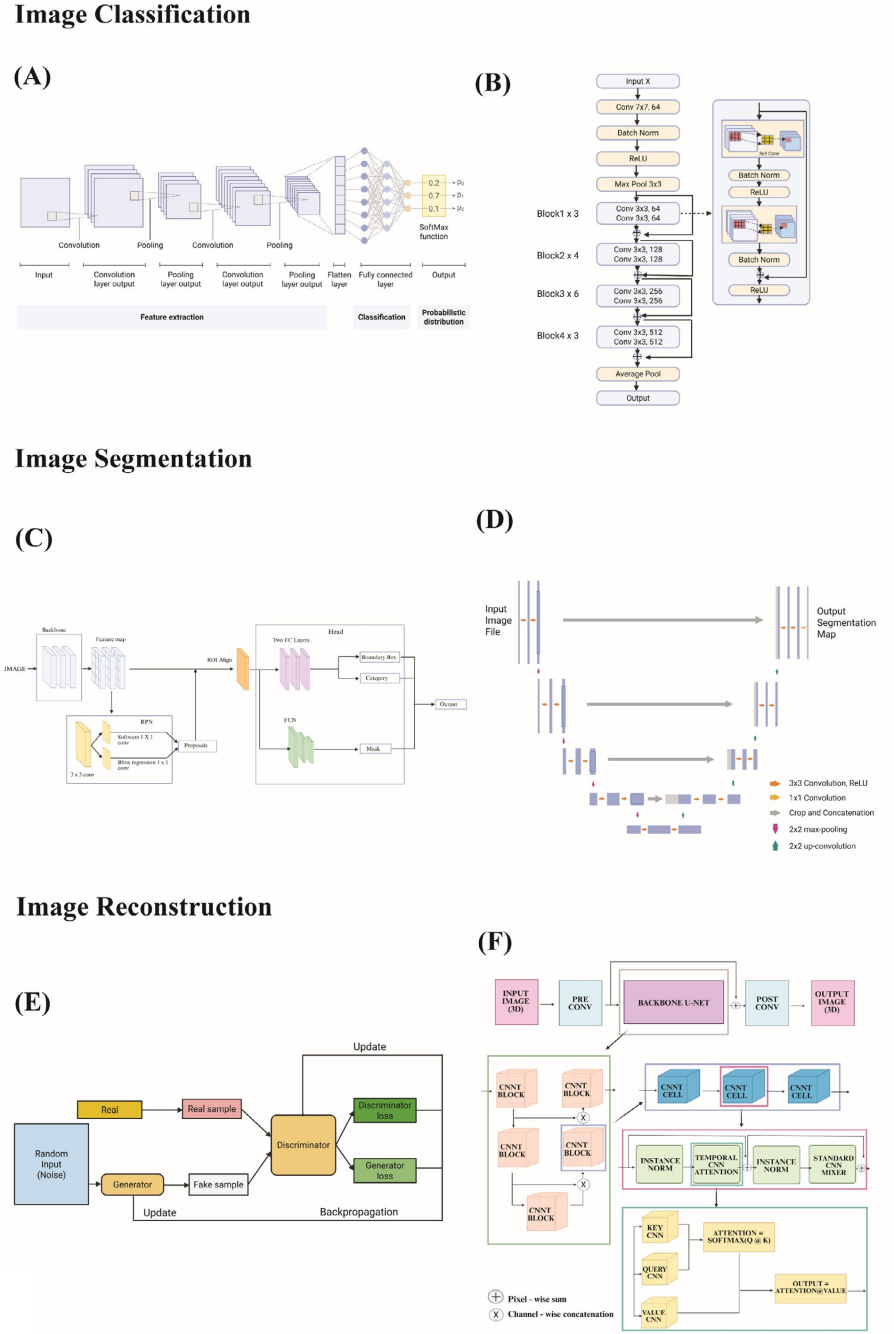
An overview diagram containing the architecture of deep learning models. (A) CNN (reproduced with permission from Takahashi et al. (2024)). (B) ResNet‐34 (reproduced with permission from Wang et al. (2025)). (C) Mask R‐CNN (reproduced with permission from Yin et al. (2024)). (D) U‐Net (reproduced with permission from Siddique et al. (2021)). (E) GAN (reproduced with permission from Sharma et al. (2024)). (F) CNNT (reproduced with permission from Rehman et al. (2024)). Each deep learning architecture has been annotated with its core features in sequence and segregated by their purpose of image classification, image segmentation and image reconstruction.

Figure 3 depicts the various architecture of DL models.

### 3.3 Image Reconstruction

Image reconstruction is a process of transforming raw data obtained from scanning an object from different angles into a complete image. This is a crucial step to be performed to remove noise, artifacts and maintain clarity in the images (Koetzier et al. 2023). The various models used to perform this task are as follows:

#### 3.3.1 GAN (Generative Adversarial Network)

GAN has two networks known as the generator and the discriminator. The generator generates fake data, and the role of discriminator is to efficiently discriminate between fake and real data (Ali, Ali, et al. 2025). Under the discriminator's guidance, the generator refines its output to generate images that more closely reflect the real data after first creating images from a random noise vector (Aalaei et al. 2023). Generative models have had great success in image processing, image style migration, and popularity density estimate. The number and dimensionality of samples are increasing at a rapid pace, and deep generative models with several hidden layers are gradually replacing generative models. Since its proposal in 2014, the GAN has emerged as a prominent area of study for deep generative models (Zhou, Li, et al. 2023). However, GAN also poses certain limitations and is still a challenging model. This model is sensitive to hyperparameters, and a good balance is required to achieve consistent gradients between the discriminator and the generator. One of the essential limitations is that the model fails to learn the different modes of data distribution resulting in samples with less variability (Lesmes‐Leon et al. 2025).

#### 3.3.2 RCAN (Residual Channel Attention Network)

The residual modules and long skip connections enable the original input to skip several levels and be reintegrated later. This enables the model to learn the changes (residual mapping) as opposed to direct mapping, which is more efficient for deep networks (Liu et al. 2024). A key concept, channel attention, refers to modulating the weights of feature channels based on their importance to the task. Using global average and max pooling, each feature channel information is extracted, and a multilayered perceptron and sigmoid activation function are used to learn its importance, followed by reweighting the feature map (Le and Kim 2023; Ye et al. 2021). The RCAN's deep architecture containing over 400 convolutional layers can limit its capability due to outdated training methods, and result in underfitting, requiring careful and high‐quality training data. Also, this residual‐in‐residual structure allows the model to focus on high frequency details of images and discard the low frequency ones (Lin et al. 2022). This reduces the influence of less significant channels and thus guides the network to prioritize critical visual features.

#### 3.3.3 CNNT (Convolutional Neural Network Transformer)

The architecture of a CNN incorporates inductive biases, such as translation invariance, which enable it to capture fine grained patterns in an image effectively. Transformers are effective at representing long‐range dependencies through their self‐attention mechanism. Thus, they can comprehend the connections between distant areas of an image (Yuan et al. 2023). They, however, individually have limitations such as the inability of CNN to capture long‐range dependencies and weak local feature extraction of transformers (Haruna et al. 2025). The objective of this combined strategy is to overcome the drawbacks of only a pure CNN or pure transformer based model and amplify the advantages of both architectures simultaneously (Chen, Wu, et al. 2024). Experiments show that use of CNNT improves quality of images and requires less time for training, outperforming other CNN‐based models, though coming with high computational costs (Rehman et al. 2024).

#### 3.3.4 ResU‐Net

ResU‐Net is a hybrid deep learning model that integrates ResNet and U‐Net models. Built upon the foundation of the U‐Net encoder‐decoder architecture, it uses the principle of skip connections (residual blocks) from the ResNet model, to simplify the process (Katsamenis et al. 2024; Liu, Dong, et al. 2022). This integration resolves the degradation issues regarding the training of models that occur with increasing network depth (Huang et al. 2025). The addition of residual block enhances the feature learning ability, and this model performs better than other models in preserving features of low‐contrast images (Liu, Kang, et al. 2022; Zhang, Zheng, et al. 2022). But this ResU‐Net model can be sensitive to gaussian noise (Huang et al. 2025). Training of this model however is expensive and time‐consuming owing to its complex and deep structure, and occasionally it may struggle to adapt to unseen data (Morotti et al. 2021).

#### 3.3.5 CARE (Content Aware Image Restoration)

CARE, a model proposed by Weigert et al. aids in obtaining the isotropic resolution of data from the anisotropic resolution of undersampled data. Even with a 60‐fold reduction in photons during capture, it is capable of restoring microscope images meaning that CARE can restore images from low signal‐to‐noise ratio inputs (Weigert et al. 2018). To train the network, image pairs of short exposure vs. long exposure (noise2 signal) are used after which the model learns to denoise in a content aware manner. CARE can resolve a key constraint in luminescence imaging—high quality restoration of images while reducing the exposure time, but like a lot of DL models are prone to artifacts (Boothe et al. 2023).

## 4 Applications

### 4.1 Conventional Optical Microscopy

**FIGURE 4:**
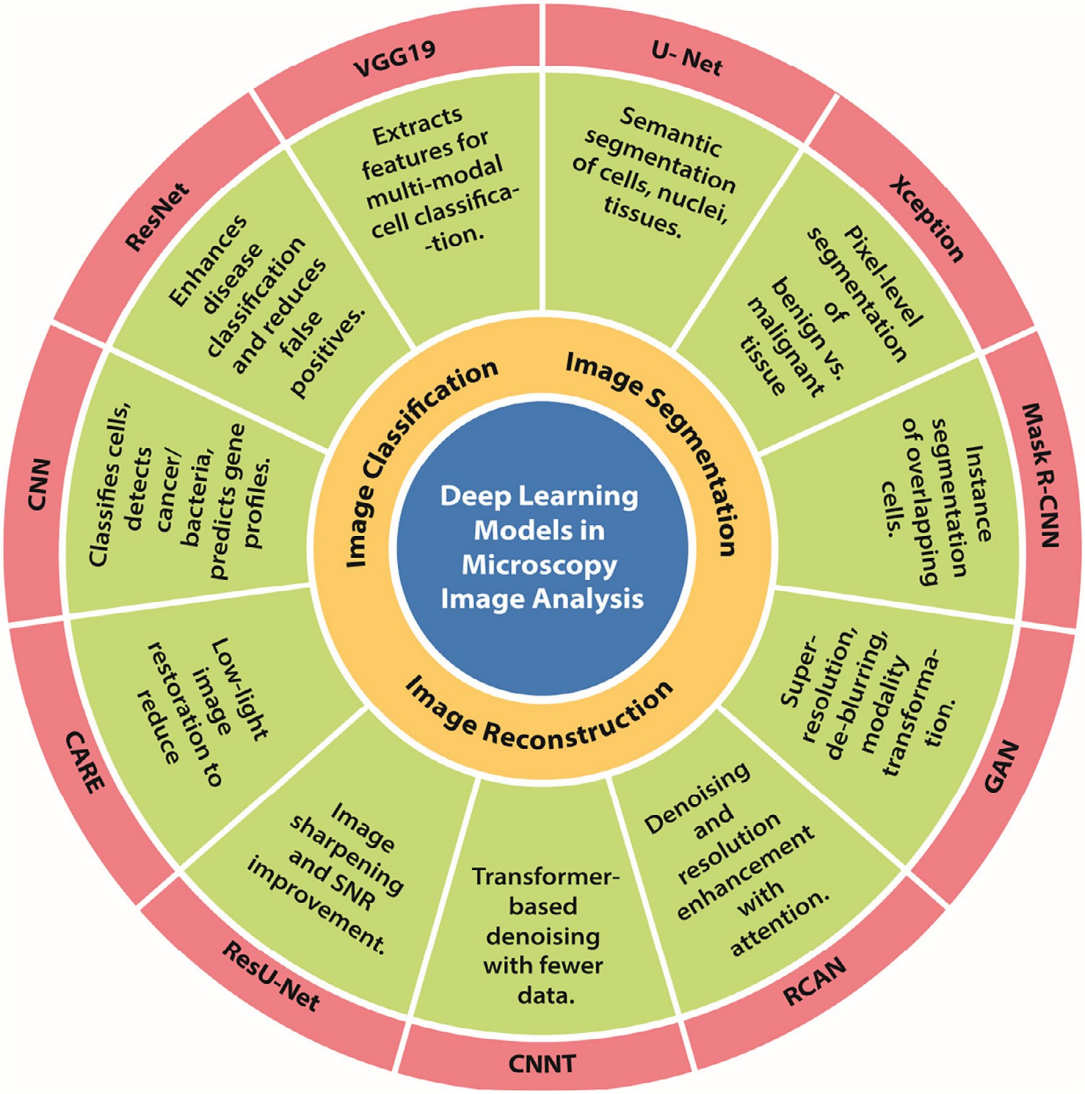
A circular schematic summarizing commonly used DL models in microscopy image analysis, mapped to their primary applications—image classification, segmentation, and reconstruction.

Brightfield microscopy images usually have low differentiation and poor contrast between the sample and background, therefore causing difficulty in image analysis. DL methods like CNN and U‐Net have advanced image classification, object detection, and segmentation (Ali et al. 2021). The incorporation of brightfield microscopy with DL models, such as CNN, has been used for the identification of white blood cells and breast cancer cells (MCF‐7) and in tuberculosis diagnosis by accurately differentiating bacilli from non‐bacilli (Greeshma and Vishnukumar 2025; Moallem et al. 2022). Similarly, another model was designed that classified eight cell lines accurately (Ferreira and Silveira 2024). The deep learning models, such as U‐Nets and their variants, have shown high potential in solving the challenges of brightfield microscopy images, such as weak contrast and poor labelling (Ghaznavi et al. 2022), with models such as YeastNet and attention‐gated U‐Nets enhancing the yeast cell segmentation and detecting the nucleus features, respectively (Ali et al. 2021; Salem et al. 2021). In addition, the Xception model has helped in detecting subtle parasitic features, which helped distinguish between parasite and host cells (Ali, Benfante, et al. 2025). A deep learning system comprising four different models was designed to predict human‐induced pluripotent cells (hiPSCs) from bright‐field microscopy images. The models were RNN for predicting colony growth, CNN for classifying the reprogramming stage, CRE‐net for identifying cell clusters, and U‐net for segmentation of cells. The integration of these four DL models allowed early detection of pluripotent cells and hence supports noninvasive and real‐time tracking (Chu et al. 2023). This study highlights that combining multiple different DL models helps researchers in investigating vulnerable and live biospecimens. Overall, the accuracy of image classification and segmentation of brightfield microscopy can be enhanced by integrating it with deep learning models, but there is still a requirement for improvement, as their efficiency may be restricted by a very noisy image or overlapping cells. Figure 4 explains the various DL models used in microscopy applications.

DIC, a label‐free microscopy technique commonly used in cell biology with low photodamage but faces common drawbacks like inadequate instance segmentation and struggles in quantitative analysis because of local gradients. Thereby highlighting the need for DL techniques specifically for DIC images (Kale et al. 2024; Pan et al. 2024). DIC microscopy integrated with CNNs has been employed for classifying molecular states by predicting morphology‐related gene expression, supporting label‐free transcriptome profiling (Jin et al. 2023), and for automatic, high‐throughput structural analysis of platelets undergoing distinct treatment (Kempster et al. 2022) and for classifying the different cell death morphologies, even in cases where molecular signals are unknown (Centofanti et al. 2025). A U‐Net‐based model such as Cellpose, segments malaria‐infected cells in DIC images, thereby assisting in monitoring the life cycle (Frangos et al. 2025). Optical flow with U‐Net provides accurate tracking of intracellular activities, such as vesicle mobility and cytoplasmic streaming (Kale et al. 2024). Advanced models combined with iterative filtering, a ResNet classifier, and the Mask R‐CNN enhance the segmentation accuracy, particularly in congested cell regions (Pan et al. 2024). To improve the Mask R‐CNN, Nguyen et al. (2024) recommended two methods: one semisupervised method that learns from both labeled and unlabeled data using a blur‐consistency framework, and an advanced preprocessing method (Nguyen et al. 2024). These studies show how DIC integrated with DL provides detailed, noninvasive, and high‐throughput cellular analysis; forthcoming studies need to focus on upgrading them for multiple cell types and dynamic cellular processes.

**FIGURE 5:**
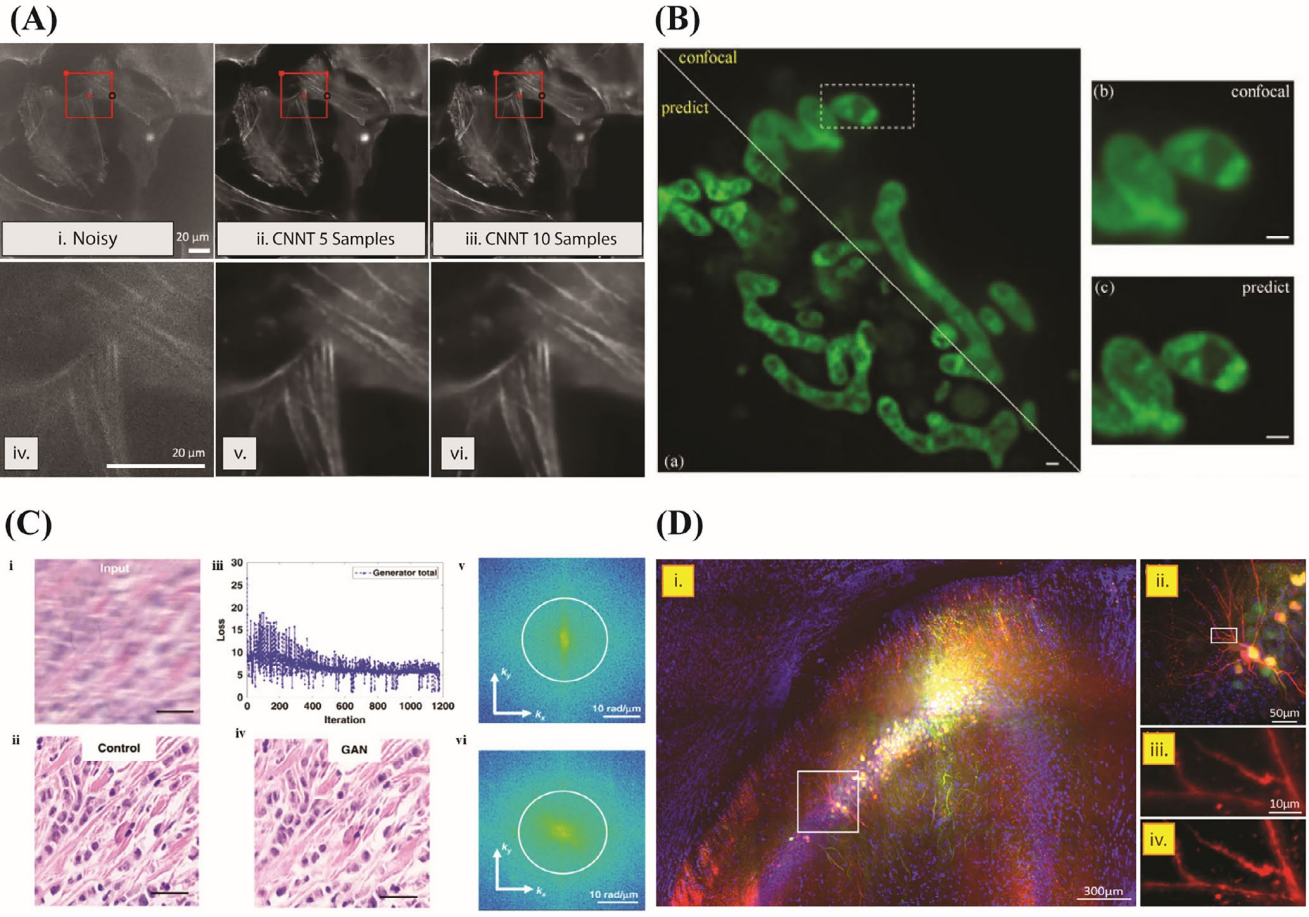
Graphical summary of outcomes of incorporating deep learning models in imaging of biological samples (A) Fluorescence microscopy images of MEF cells. (i) noisy input image; (ii—iii) CNNT results after 30 epoch finetuning on 5 and 10 samples; (iv—vi) Enlarged view of red box region (reused with permission from Rehman et al. (2024)). (B) Deep‐learning prediction of mitochondrial structures in live cells. (i) confocal vs. model prediction; (ii—iii) Enlarged view of dotted yellow box region (reused with permission from Cui et al. (2024)). (C) Image processing process of GANscan: (i) input blurred image; (ii) control image; (iii) loss curve of GAN training; (iv) GAN‐restored output; (v and vi) frequency spectra representing the recovery of lost high‐frequency content (reused with permission from Fanous and Popescu (2022)). (D) Airy beam light sheet imaging of mouse brain (Mb2) labeled with Hoechst (blue), YFP (green), and tdTomato (red). (i) Deconvolved whole‐sample projection; (ii) Zoom of i; (iii) Raw tdTomato; (iv) Deconvolved tdTomato (reused with permission from Stockhausen et al. (2023)).

PCM, specifically label‐free phase contrast imaging has benefited from deep learning methods. CNNs and vision transformers have been employed to classify bacterial species from time‐lapse data, demonstrating the capability to distinguish fine morphological differences (Hallström et al. 2023). Additionally, a self‐supervised segmentation algorithm trained directly on raw imaging data across multiple modalities—including phase contrast—has achieved performance comparable to supervised techniques (Lam et al. 2025). While the results are promising, many approaches are developed and tested on limited datasets. Expanding their scalability and integration with downstream analytical workflows is key to advancing clinical relevance. Among these approaches, U‐Net‐based architectures excel in label‐free segmentation of subcellular structures in high‐resolution images. Combined 2D/3D U‐Nets have been used for segmenting nuclei and mitoses in stem cell colonies using fluorescence‐based labels (Asmar et al. 2024). U‐Nets also reconstruct phase maps from defocused bright‐field images (Wang, Ali, et al. 2023). To address imaging artifacts like halos and shadows, physics‐informed and self‐supervised U‐Nets have shown promise (Shimasaki et al. 2024). However, dependence on paired training data can limit scalability in more diverse or dynamic imaging conditions. To address image reconstruction challenges in phase‐contrast microscopy, GANs have enabled significant improvements in phase‐contrast microscopy by accelerating image reconstruction while preserving fine structural details. GANscan, a pix2pix‐style model, effectively deblurs continuously scanned images by learning from blurred–sharp pairs, enhancing edge recovery and acquisition speed (Fanous and Popescu 2022) (as shown in Figure 5). More recently, Thapa et al. (2024) introduced a conditional GAN for single‐shot phase retrieval, replacing multi‐frame TIE‐based methods and maintaining high fidelity. The GANscan approach utilizes a GAN to deblur continuously scanned phase‐contrast images. Alternative methods such as UNN‐DPC explore reconstruction without labeled data using untrained networks (Seong et al. 2023). These developments show that GANs can rapidly generate high‐quality phase‐contrast images, but they remain sensitive to training data diversity and domain shifts, limiting generalizability.

### 4.2 Fluorescence Microscopy

The highest‐quality diffraction‐limited imaging of fluorescence microscopy images is only possible in the absence of optical aberrations. However, these aberrations are dominant and cause three‐dimensional (3D) images to deteriorate in terms of the signal‐to‐noise ratio (SNR), contrast, and resolution. The traditional techniques used to remove these aberrations, termed adaptive optics (AO), use wavefront sensing to correct the distorted wavefront by applying a corrective wavefront (Guo et al. 2025). The residual channel attention network (RCAN) DL model, trained on fluorescence microscopy data, effectively denoises and aides in acquiring images without photobleaching. Compared with other models, RCAN outperforms CARE, SRResNet, and ESRGAN in terms of resolution enhancement (Chen et al. 2021). Compared with AO techniques, the ‘DeAbe’ RCAN model improved the SNR and restored resolution at greater depths in the tissues of *Caenorhabditis elegans* embryos (Guo et al. 2025). Currently, RCAN relies on supervised training that needs paired high‐ and low‐quality images. Improvements such as the incorporation of framework like Noise2Noise would enable the model to reconstruct images without needing a ground truth reference image. Though RCAN's capability has been shown in *C. elegans* embryos, its performance across diverse fluorophores and sample types is as yet unexplored and requires evaluation. Another approach, the convolutional neural network transformer (CNNT) model, which combines CNN and transformer networks to improve denoising, has been established. It surpasses the 3D‐RCAN model because it possesses equal image quality, and better fine‐tuning with fewer image‐volume pairs (Rehman et al. 2024). The fusion approach of CNNT provides it with a greater advantage compared to other DL models. However, the incorporation of transformers makes it computationally expensive as they require more memory. Research into lightweight attention mechanisms could allow the model to run faster.

Conventional confocal microscopy imaging with a resolution of approximately 180 nm needs improvement for more accurate observations of biological samples. Traditional methods such as confocal subtraction imaging, and the Richardson–Lucy deconvolution algorithm slightly increase the resolution. Deep learning methods such as Res U‐Net significantly improved the resolution to approximately 120 nm in a cost‐effective manner and clearly distinguish mitochondrial ridge structures, which otherwise was not possible (Cui et al. 2024). ResU‐Net was shown to significantly increase SNR, thereby increasing the resolution of image samples of mouse cardiac tissue (Suresh et al. 2025). The above studies demonstrate that hybrid models such as ResU‐Net are able to extract the advantages of each model it comprises of. Moreover, future models that combine ResU‐Net's method of learned upsampling along with Richardson–Lucy deconvolution could improve the resolution beyond 120 nm, through utilization of both data‐driven patterns and point‐spread functions.

Widefield is one of the prominent fluorescent imaging techniques which is less invasive and essential for imaging applications. This technique generated ultrawidefield images which were used by DL model ResNet‐101 to classify and screen fundus disease, this could be helpful especially in places where there is a lack of ophthalmologists. It was used with UWFIs to create an automated screening tool to circumvent human resource constraints (Sun et al. 2023). This DL model works well compared to the doctors in diagnosing the disease but since it was trained on a small dataset, it might not work well if the images are fed from different sources. Realistic ultra‐widefield (UWF) retinal images are produced by GANs for diseases such as retinitis pigmentosa and diabetic retinopathy. These artificial images improve the performance of deep learning models for disease detection by supplementing small datasets (Lei et al. 2023). GAN manages to produce images with limited datasets but there can be inconsistency depending on the quality of input data which can lead to poor image resolution or loss of details and eventually misinterpretation.

Live cell images were generated using multiple microscopy techniques such as TIRF, SDC and phase contrast microscopy. These images were segmented using MARS‐Net pipeline consisting of VGG19 encoder, U‐Net decoder and dropout layers (Jang et al. 2021). This model performs well with isolated cells but in case of overlapping cells this model can fail to classify, it can either mislabel it or fail to even detect it.

### 4.3 Nonlinear Multiphoton Microscopy

**FIGURE 6:**
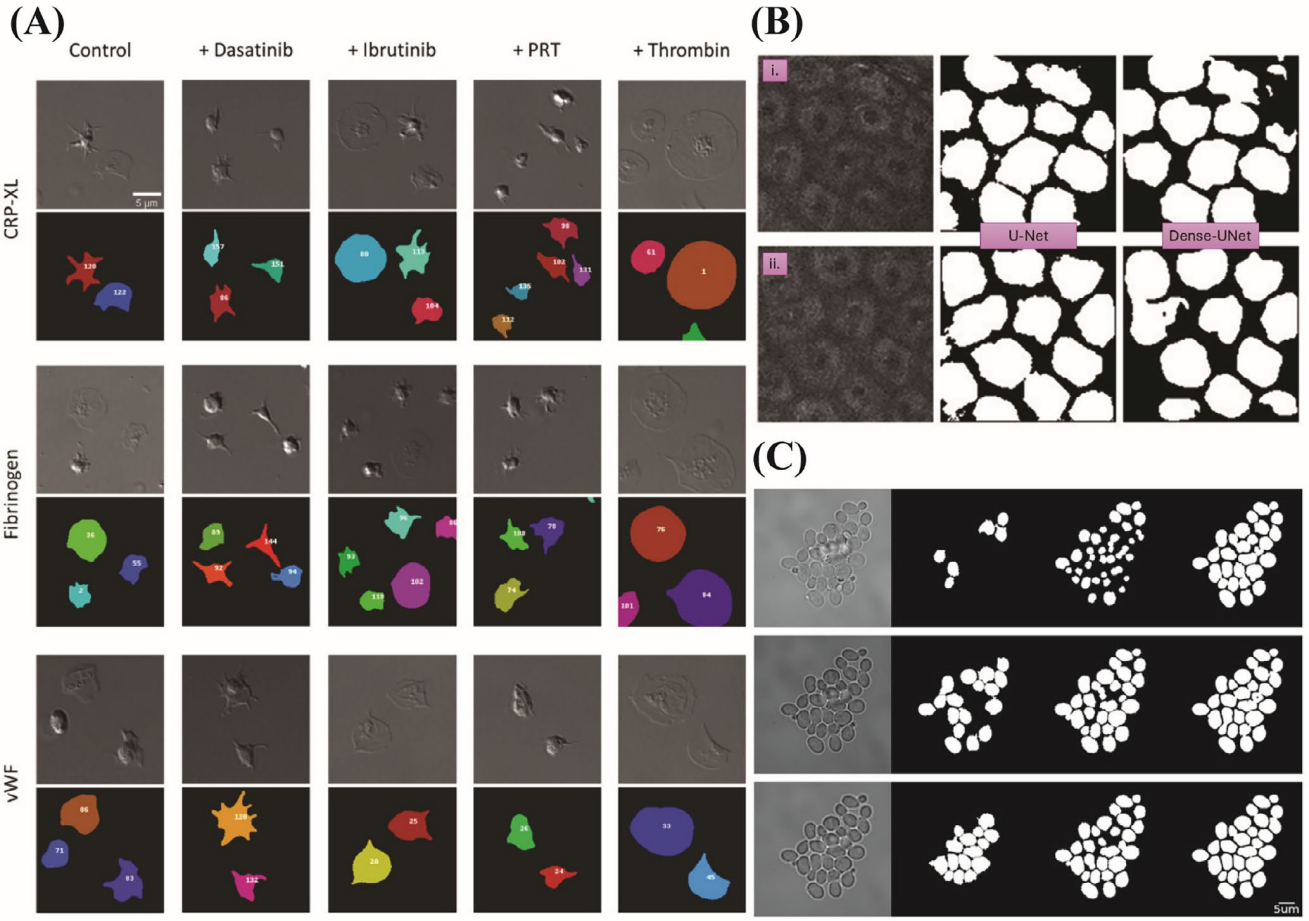
Compilation of deep learning model applications in biological sample imaging (A) Automated segmentation and classification of platelet structures obtained by DIC images using CNN (reused with permission from Kempster et al. (2022)). (B) Multiphoton images of human skin in vivo and segmentation results. (i—ii) represent images of human skin in vivo and next columns represent segmentation using U‐Net and Dense‐UNet models respectively (reused with permission from Cai, Tian, et al. (2020)). (C) Comparison of cell segmentation at different level of focus. Among three YeastNet provides better image segmentation than Cell Star and non‐trainable method (reused with permission from Salem et al. (2021)).

Nonlinear multiphoton microscopy is essential for the high‐resolution imaging of biological tissues. Improvements can be made, and inconsistencies in the data can be avoided with the help of DL models (Lu et al. 2025). The U‐Net model performs image segmentation to extract elastic fibers and cells from images, allowing distinct separation of pathologically important sections from normal (Wang, Huang, et al. 2023). Cai, Tian, et al. (2020) demonstrated that the Dense‐U‐net model is able to segment in vivo skin cell images with higher precision than regular U‐Net. Figure 6 shows how DL models are used in multiphoton imaging. U‐Net requires a large amount of high‐quality training data but to acquire such data challenges such as contrast loss at tissue depths need to be overcome. Also, it should be noted that though the Dense‐UNet model has higher precision, it comes at the cost of increased memory and computational power, necessitating optimization in future. A CNN with the Xception architecture was used to separate malignant colon tumors from healthy or benign colon tissues (Terradillos et al. 2021). Because Xception was designed to be efficient and have fewer parameters, it may reduce its capability of understanding how different image features relate to each other, or inter‐channel interactions. Introduction of attention modules could help the model learn which features to focus on. Evaluating its performance on other microscopy types would help us understand its potential for generalized applications. The phototoxicity of tissues necessitates lower exposure to light, resulting in low resolution images. Deep learning methods such as CARE successfully reconstruct high resolution images from the lower resolution images obtained, demonstrating the application of DL to reduce harmful light exposure while maintaining high resolution and improving the safety of imaging the retina of eyes (McAleer et al. 2021). The success of CARE in denoising from low‐resolution images is impressive and sets it apart from other models. Integrating its use as a denoiser as a preprocessing step along with downstream image segmentation models like U‐Net or Xception would result in an efficient pipeline.

Second Harmonic Generation (SHG) images with multipolarization were used to train the ResNet model to classify and measure collagen type I and type II mixes in hydrogels. This approach is relevant for examining osteoarthritis, which is associated with variations in collagen composition within cartilage (Nair et al. 2023). It is a brilliant model, but it could have been trained on a large dataset with 3D images and data so that it can accurately study the images and perform better.

Coherent Raman scattering (CRS) microscopy, encompassing the techniques stimulated Raman scattering (SRS) and coherent anti‐Stokes Raman scattering (CARS), enables rapid, label‐free imaging. Broadband SRS, for example, can acquire a full Raman spectrum at each pixel (la De Cadena et al. 2022), greatly enhancing chemical specificity by retrieving rich hyperspectral data. Deep learning has been key in translating these images into diagnostic insights. Deep learning techniques like CNNs, have demonstrated considerable promise in facilitating swift and label‐free histopathological analysis using stimulated Raman scattering (SRS) microscopy. Research has substantiated their efficacy in differentiating between cancerous and healthy tissues and in conducting Gleason grading without reliance on traditional staining techniques (Ao et al. 2023; Weber et al. 2024). Notably, the introduction of techniques such as VQSRS, a self‐supervised network grounded in variational quantization‐based variational autoencoders (VQ‐VAE), achieves performance levels comparable to conventional CNNs while significantly expediting image analysis (Wang et al. 2024). Nonetheless, challenges persist, including the necessity for extensive annotated datasets and diminished performance when addressing rare tumor categories. Future research should focus on harnessing unsupervised and transfer learning to enhance the robustness of these techniques under varying conditions. In parallel, U‐Nets have been effective in segmentation, denoising, and spectral unmixing of SRS data. Even with minimal training pairs, basic U‐Nets outperform classical filters for recovering intracellular features (Abdolghader et al. 2021). Self‐supervised (Noise2Noise) and unsupervised (UHRED) models further reduce the need for labeled datasets, supporting chemical segmentation (Abdolghader et al. 2021). Semantic segmentation of tumor cells has been achieved using U‐Nets with postprocessing to yield accurate instance masks (Zhang, Yun, et al. 2021). More advanced architectures like U‐within‐U process both spatial and spectral information in hyperspectral data (Zhou et al. 2025), and U‐Nets combined with VISTA expansion imaging have enabled label‐free histological mapping of brain structures (Tipping et al. 2024). Remaining challenges include domain variability, data dimensionality, and annotation costs, driving interest in hybrid and semi‐supervised approaches for broader clinical adoption.

CNNs have proven effective for analyzing CARS hyperspectral data. The CSAN model, for example, has been used to classify amyloid‐β plaques in Alzheimer's disease models (Luo et al. 2023), and transforming 1D spectra into 2D image representations has further improved CNN‐based classification (Luo et al. 2023). The spectral richness of broadband CARS imaging supports its growing role in cellular diagnostics, although broader validation and integration with spectral‐domain learning remain essential for wider clinical adoption. In addition to CNN‐based techniques, GANs are increasingly used for non‐resonant background removal and spectral denoising, though their reliability depends strongly on training data diversity. Vernuccio et al. (2024) applied a GAN‐based encoder–decoder to broadband CARS spectra, demonstrating improved peak preservation and real‐time processing. Luo, Xu, et al. (2024) trained a GAN on synthetic noisy spectra to subtract background in real samples, enabling label‐free recovery of chemical features. While these approaches avoid explicit physical modeling, their reliability hinges on how well training data represents true spectral distributions. These highlight GANs' potential in hyperspectral CARS imaging but caution that they may hallucinate peaks or fail when confronted with new chemical contexts.

### 4.4 Smartphone‐Based Microscopy

The smartphone‐based microscopy, integrated with DL models, is transforming image analysis in both medical diagnosis and environmental applications. A CNN‐based model such as SSD‐Mobile Net has been employed for the automatic detection of *Trichuris trichiura* eggs by analyzing Kato–Katz images (Dacal et al. 2021). Recently, another model, YOLOv5, was designed for automatic identification and quantification of microplastics (Bin Zahir Arju et al. 2025). A CNN‐based smartphone model was used for automatic detection of malaria parasites in thick blood smears and used in diagnosis and differentiating the subtypes of Microbial Keratitis (MK) (Soleimani et al. 2025; Yang et al. 2020). The integration of smartphone‐based model with DL model such as EGE‐UNet is used in non‐invasive hemoglobin prediction and CycleGAN is used in enhancing the image quality (Chen, Hu, et al. 2024; Zhang, Zhang, et al. 2024). These methods are portable and efficient in analysis, but still, there is a need to focus on further studies as they face limitations like restricted training data and image variability, thereby making them more accurate and reliable.

### 4.5 Light Sheet Microscopy

The U‐Net architecture, along with its 3D adaptations, has become a popular choice for analyzing LSFM data. Its use of skip connections allows the model to retain spatial details across multiple scales, which is especially useful for segmenting intricate features like zebrafish vasculature (Yin et al. 2022). More recent innovations, such as UI‐Trans, build on this foundation by incorporating Vision Transformers. This addition helps the model recognize long‐range relationships in the data, leading to more accurate segmentation—even in difficult scenarios like cardiac images affected by light scattering (M. Zhang et al. 2025). Despite these advances, segmentation is still challenged by anisotropic resolution, uneven illumination, and limited labeled data—issues that domain adaptation and attention mechanisms may help overcome. GANs have likewise expanded LSFM's capabilities, particularly for deconvolution and super‐resolution. Wijesinghe et al. (2022) used an unsupervised GAN trained on simulated PSF‐blurred data to deconvolve engineered beams, improving contrast without paired ground truth (Wijesinghe et al. 2022). Stockhausen et al. (2023) deployed a conditional GAN to translate low‐NA “pencil” beam images into high‐contrast equivalents, enhancing resolution while maintaining throughput (Stockhausen et al. 2023). These models typically use U‐Net or attention‐based generators with physics‐informed losses. However, they are system‐specific and often require retraining for new optics or sample types. Wagner et al. (2021) demonstrated a hybrid deep‐learning system combining light‐field and light‐sheet inputs to enable video‐rate 3D reconstruction (Wagner et al. 2021). Overall, GANs offer powerful enhancements for LSFM, but care must be taken to avoid overfitting, manage training data requirements, and validate against artifacts.

Table 1 provides a comprehensive summary of all the applications discussed in our review. For each microscopy modality, the corresponding deep learning models and their specific applications are listed to clearly reflect the scope and coverage of the studies analyzed.

**TABLE 1:** DL models and their applications across various optical microscopy techniques.

Microscopy technique	DL model	Applications	References
TIRF	Mars‐Net	To segment live cell images accurately	(Jang et al. 2021)
Widefield	ResNet	To develop an automated fundus disease screening tool	(Sun et al. 2023)
	GAN	Produces realistic images for disease detection	(Lei et al. 2023)
SHG	ResNet	To classify and measure collagen types for examining osteoarthritis	(Nair et al. 2023)
Multiphoton	U‐Net CNN	Extraction of elastic fibers and cells from images to identify important regions	(Wang, Huang, et al. 2023)
	Dense‐UNet	Higher precision segmentation in vivo skin cell images	(Cai, Tian, et al. 2020)
	CARE	Reconstruct high resolution image from low resolution reducing phototoxicity	(McAleer et al. 2021)
Fluorescence	RCAN	Restored resolution of *C. elegans* embryo tissues	(M. Guo et al. 2025)
	CNNT	Improvement of quality of image through denoising	(Rehman et al. 2024)
Confocal	ResUNet	Improve resolution cost‐effectively	(Cui et al. 2024)
		Increase resolution in mouse cardiac tissue	(Suresh et al. 2025)
Brightfield	CNN	Label‐free, accurate classification of different cell types, such as cancer cells, TB bacilli, and white blood cells	(Ferreira and Silveira 2024; Greeshma and Vishnukumar 2025; Moallem et al. 2022)
	Xception	Distinguish parasite and host cells by detecting accurate features	(M. Ali, Benfante, et al. 2025)
	U‐Net	Robust segmentation of individual cells, organelles, and nuclei in images with poor labeling or low contrast, including precise MCTS segmentation and semantic segmentation for label‐free live‐cell imaging	(Ali et al. 2021; Chu et al. 2023; Ghaznavi et al. 2022; Salem et al. 2021; Streller et al. 2025)
	CRE‐Net	Refining CNN output and detecting compact cell clusters	(Chu et al. 2023)
	RNN	Predicting future colony growth	(Chu et al. 2023)
DIC	Mask R‐CNN	Real‐time, non‐invasive cell analysis as well as high‐precision, label‐free segmentation of HepG2 cell adhesion in DIC microscopy images	(Nguyen et al. 2024; Pan et al. 2024)
	CNN	Classification of cell death morphologies from DIC images and nondestructive prediction of transcriptome profiles (MCGs).	(Centofanti et al. 2025; Jin et al. 2023)
	U‐Net	Tracking intracellular dynamics (cytoplasmic streaming, vesicle movement)	(Kale et al. 2024)
	ResNet	Classification and false‐positive filtering in segmented DIC cell images	(Pan et al. 2024)
Smartphone‐Based microscopy	CNN	Automated quantification of parasite eggs (SSD‐MobileNet), identification of microplastics (YOLOv5), automatic detection malaria parasite and in diagnosis and differentiating subtypes of microbial keratitis	(Bin Zahir Arju et al. 2025; Dacal et al. 2021; Soleimani et al. 2025; Yang et al. 2020)
	EGE‐UNet	Non‐invasive prediction of hemoglobin	(Chen, Hu, et al. 2024)
	CycleGAN	Enhances the image segmentation	(Zhang, Zhang, et al. 2024)
Phase Contrast Microscopy	CNN, ViT	Bacterial species ID, morphology classification	(Hallström et al. 2023)
	U‐Net, ConvNeXt U‐Net	Nuclei/mitosis segmentation, subcellular segmentation, phase map recovery	(Asmar et al. 2024)
	GAN	Phase retrieval, deblurring, emerging 3D tomogram reconstruction, super‐resolution	(Fanous and Popescu 2022)
CRS Microscopy	CNN (CSAN)	Amyloid‐β plaque detection, spectral classification	(Luo et al. 2023; Zhou, Zhang, and Xu 2023)
	GAN, Encoder‐Decoder GAN	Spectral denoising, non‐resonant background removal	(Luo, Xu, et al. 2024; Vernuccio et al. 2024)
	CNN, VQ‐VAE (VQSRS)	Cancer classification, Gleason grading, label‐free histopathology	(Ao et al. 2023; Wang et al. 2024)
	U‐Net, U‐within‐U	Tumor segmentation, intracellular structure segmentation, spectral unmixing	(Abdolghader et al. 2021; Zhang, Hu, et al. 2023)
LSFM	3D U‐Net, UI‐Trans	Embryo, organoid, vasculature segmentation, spatial structure capture	(Yin et al. 2022; Zhang et al. 2025)
	GAN, Hybrid models (GAN + CNN)	Super‐resolution, beam deconvolution, 3D reconstruction, 3D video‐rate reconstruction	(Wagner et al. 2021; Wijesinghe et al. 2022)

## 5 Challenges

Deep learning works well in the field of microscopy because experimental datasets are usually collected in extremely controlled settings, and it has been used to create task‐specific models for microscopy image classification, segmentation, tracking, and super resolution. Despite its benefits, there are still significant obstacles (De Haan et al. 2020; Liu et al. 2021). High‐quality data is essential for machine learning performance, therefore careful pre‐processing such as eliminating outliers, missing values, and unnecessary features is essential for precise predictions. Nevertheless, this procedure can be laborious and intricate, and mistakes in pre‐processing could still produce inaccurate outcomes (Amin et al. 2024). Large, domain‐relevant pretraining datasets are essential for the generalization of deep learning models. Using mismatched datasets can result in overfitting, inadequate accuracy and negative transfer in specialized domains like remote sensing or medicine (Van Tilborg et al. 2024). When it comes to evaluating medical imaging data, DL models like CNNs have shown impressive performance. The requirement for large and varied datasets, as well as the difficulty of generalizing across various imaging modalities and acquisition techniques, are obstacles they must overcome (Alsubaie et al. 2024). Additionally, it could be challenging to obtain data with a balanced distribution for every subgroup, which could lead to biased statistics. When DNNs are trained on biased data, they may produce inaccurate predictions (156) (Tian et al. 2021). The surge in bioimaging data over the last 10 years has increased the need for sophisticated analysis tools (von Chamier et al. 2021).

## 6 Conclusion

Optical microscopy plays a vital role in biomedical applications, offering high resolution and non‐invasive images, though it has drawbacks like blurring, poor resolution, and low SNR. Over the years variety of microscopes have been developed to tackle imaging challenges, few of which are brightfield, phase contrast, multiphoton, DIC, TIRF, each technique having its own advantages and drawbacks. To meet the rising demand in biomedical imaging, it is important to address these challenges and integrate approaches like deep learning to advance imaging and data interpretation. DL models, which draw inspiration from the cognitive functioning of the human brain, excel at automatically extracting complex features through their multiple hidden layers, making them an effective tool to overcome the barriers of traditional image analysis techniques. Table 1 shows the various types of DL models used in microscopy applications. In this review, we categorized the various DL models used on the basis of their functional contribution to improvement of the quality of the image, such as classification, segmentation, restoration. With the use of DL models, image classification has notably improved. CNNs are good at extracting spatial patterns for image recognition, ResNet prevents degradation in performance as network depth increases, VGG19 is easy to implement with its uniform layers, and ANNs provide the ability to connect complex nonlinear relationships to the correct output class. The accuracy of image segmentation across a range of microscopy techniques has been significantly enhanced by the integration of deep learning models such as U‐Net, Mask R‐CNN, and Xception. U‐Net excels at precise pixel‐level segmentation using minimal data, Xception provides efficient feature extraction for lightweight models, and Mask R‐CNN allows instance‐aware segmentation by accurately separating overlapping structures. Incorporation of image reconstruction models like GAN, RCAN, CNNT, ResU‐Net, and CARE has significantly improved the image quality across a range of microscopic modalities leading to faster, safer and more precise imaging. Deep learning models still have significant limitations because of domain‐specific data scarcity, pre‐processing complexity, and poor generalization across modalities, even with the controlled nature of microscope imaging. A promising direction would be to integrate these various task‐specific models to create a generic multipurpose model.

## Author Contributions

**Pottumarthy Venkata Lahari, Sagnika Dutta, H. Deeksha** and **Samreen A. Patel:** literature search and data curation, writing – original draft, review and editing. **Budheswar Dehury:** conceptualization, supervision. **Nirmal Mazumder:** conceptualization, supervision. All authors have reviewed and approved the final manuscript.

## Funding

We thank the ASEAN‐India Science & Technology Development Fund (AISTDF), Anusandhan National Research Foundation (ANRF), Government of India, India (Project Number‐ANRF/F/84/2025‐2026), and the Indian Council of Medical Research (ICMR) (Project Number‐Dev/SG‐00170/2024) Government of India, India, for financial support.

## Ethics Statement

The authors have nothing to report.

## Consent

The authors have nothing to report.

## Conflicts of Interest

The authors declare no conflicts of interest.

## Data Availability

The data that support the findings of this study are available from the corresponding author upon reasonable request.
